# Using systems thinking to understand how the South West - School Health Research Network can improve adolescent health and well-being: A qualitative process evaluation

**DOI:** 10.1016/j.healthplace.2023.103034

**Published:** 2023-04-28

**Authors:** Emily Widnall, Patricia N Albers, Lorna Hatch, Georgina Hopkins, Judi Kidger, Frank de Vocht, Eileen Kaner, Esther MF van Sluijs, Hannah Fairbrother, Russell Jago, Rona Campbell

**Affiliations:** 1 Population Health Sciences, University of Bristol; 2 Faculty of Medical Sciences, Newcastle University; 3 MRC Epidemiology Unit, University of Cambridge; 4 Health Sciences School, University of Sheffield; 5 Centre for Exercise Nutrition & Health Sciences, School for Policy Studies, University of Bristol

**Keywords:** school health research network, adolescents, health, mental health, well-being, systems-based intervention, complex adaptive systems

## Abstract

Schools offer a valuable setting to promote good health and mental well-being amongst young people. Schools are complex systems and such systems interventions are needed to improve pupil health and well-being. This paper presents a qualitative process evaluation of the South West- School Health Research Network, a systems level intervention. The evaluation is based on interviews with school staff, local authorities and wider stakeholders. Given the complexity of England’s educational system there is a need to intervene and monitor health at multiple levels and to ensure close partnership working to effectively improve adolescent health through schools.

## Introduction

Adolescence is a key point in the lifecourse in which to promote health and well-being. Risk taking behaviours increase during adolescence (Kipping et al., 2012), with adolescents having increased control and agency over their own life (Daddis, 2011) and mental health difficulties continue to rise over time with now one in six adolescents in the UK reporting a probable mental disorder, an increase from one in nine in 2017 (Newlove-Delgado, 2021). Schools are a valuable setting for both health research and health improvement amongst young people, with existing literature confirming the impact a school has on student health (Bonell et al., 2013).

Secondary schools vary greatly in how far they prioritise student health and well-being. A potential justification for not prioritising health and well-being in schools is that the more time spent on it, the less time there is for academic learning, resulting in lower attainment; a ‘zero-sum game’ (Bonell et al., 2014). However, there is a strong evidence base demonstrating that education and health are synergistic; healthy young people are better learners (Bradley and Greene, 2013) and educational attainment is associated with living a longer, healthier life. The role of schools is particularly important for adolescent health given the increased sensitivity to peer influences and increases in risk-taking behaviours at this age (Bonell et al., 2019). Additionally, school connectedness (the quality of students’ engagement with peers, teachers and learning in the school environment) has been suggested as a novel target for the prevention of depression and anxiety in adolescents (Raniti et al., 2022).

There have been a number of policies and strategies to improve health in schools. For example, to encourage educational and health institutions to coordinate efforts to promote health through schools, The World Health Organization established the Health Promotion Schools Framework (HPS), which advocates a whole-school approach (WHO, 1997). However, there are still many unknowns to the HPS Framework and its full potential remains unevaluated (Langford et al., 2015, Langford et al., 2017). In 1998, England introduced The National Healthy Schools Programme (NHSP) which aimed to encourage closer working between health and education providers. Under this scheme schools could work toward an award to achieve ‘Healthy School Status’ by performing well in personal, social and health education, healthy eating, physical activity and emotional health and well-being (Barnard et al., 2009). However, this scheme came to an end in its National remit in 2011. Despite this, a number of local authorities still use healthy schools initiatives across England which encourage schools to work towards a range healthy school awards. A key challenge is that the local authority initiatives are now all independent schemes and awards with no national criteria or standardization.

Despite existing frameworks and initiatives (Hunt et al., 2015, Stirling and Emery, 2016), several challenges remain regarding health improvement in school settings, even when schools are supportive of it, difficulties comprise intervention delivery, sustainability and cost-effectiveness and importantly, a lack of integration between academic, policy, practice and public communities to facilitate co-production of school health improvement research (Langford et al., 2017, Murphy et al., 2021).

Public health interventions are often complex and move beyond conventional thinking of interventions as a ‘package’ of activities (Hawe et al., 2009). Preventive interventions in schools ideally focus on the dynamic properties of the context they are set in and are thought of in terms of how the school operates within a wider complex system. Systems thinking conceptualises the interrelationships between components of a system (such as an individual school and the broader education system it sits within) and their relationships with the system as a whole (Trochim et al., 2006). Systems approaches are being advocated to address complex challenges in public health and can be of particular value in connecting and synthesizing the distinct strands or structures in place, helping to identify strategies for intervention sustainability, scalability, and reach (Huang et al., 2011). Schools can be conceptualised as complex adaptive systems in their own right (Keshavarz et al., 2010), but schools also sit within a broader educational and health system.

The English secondary education system (ages 11-16 years) is particularly complex with a wide range of school types including comprehensive (without reference to ability), grammar (selective based on academic ability) and fee-paying schools. Additionally, some secondary schools are managed via a local authority and others, including academies and free schools, are run by academy trusts. Academy schools in England are publicly funded state schools that run independently of local authorities and benefit from greater freedoms around the delivery of the curriculum, length of terms, school days and staff conditions. The UK government introduced the academies programme through the Learning and Skills Act 2000, in an attempt to improve pupil performance and address underperforming schools by offering a private sponsor. A recent qualitative study highlighted the varied nature of health promotion in academy schools (Jessiman et al., 2019), particularly given the absence of national policy or guidance around health promotion in schools prior to the recent statutory guidance on relationship, sex and health education (DfE, 2019). Variation in how schools in England are managed feeds into variation in the time, resources and strategies devoted to student health and well-being.

Despite an increase in the use of systems based approaches in public health research, there remain limited examples of applying systems thinking to school health promotion interventions. A recent systematic review of whole systems approaches to obesity and other complex public health challenges found very few UK based studies (n=13) and a lack of school-based system approaches (Bagnall et al., 2019). Of the school-based studies, the majority focused on primary schools. Further research is therefore required to better understand the unique systems involved within the secondary education landscape. Secondary school contexts and environments differ greatly from primary schools and health challenges faced by students will also differ, particularly regarding risk-taking behaviour, peer relationships and mental health.

**Figure 1:**
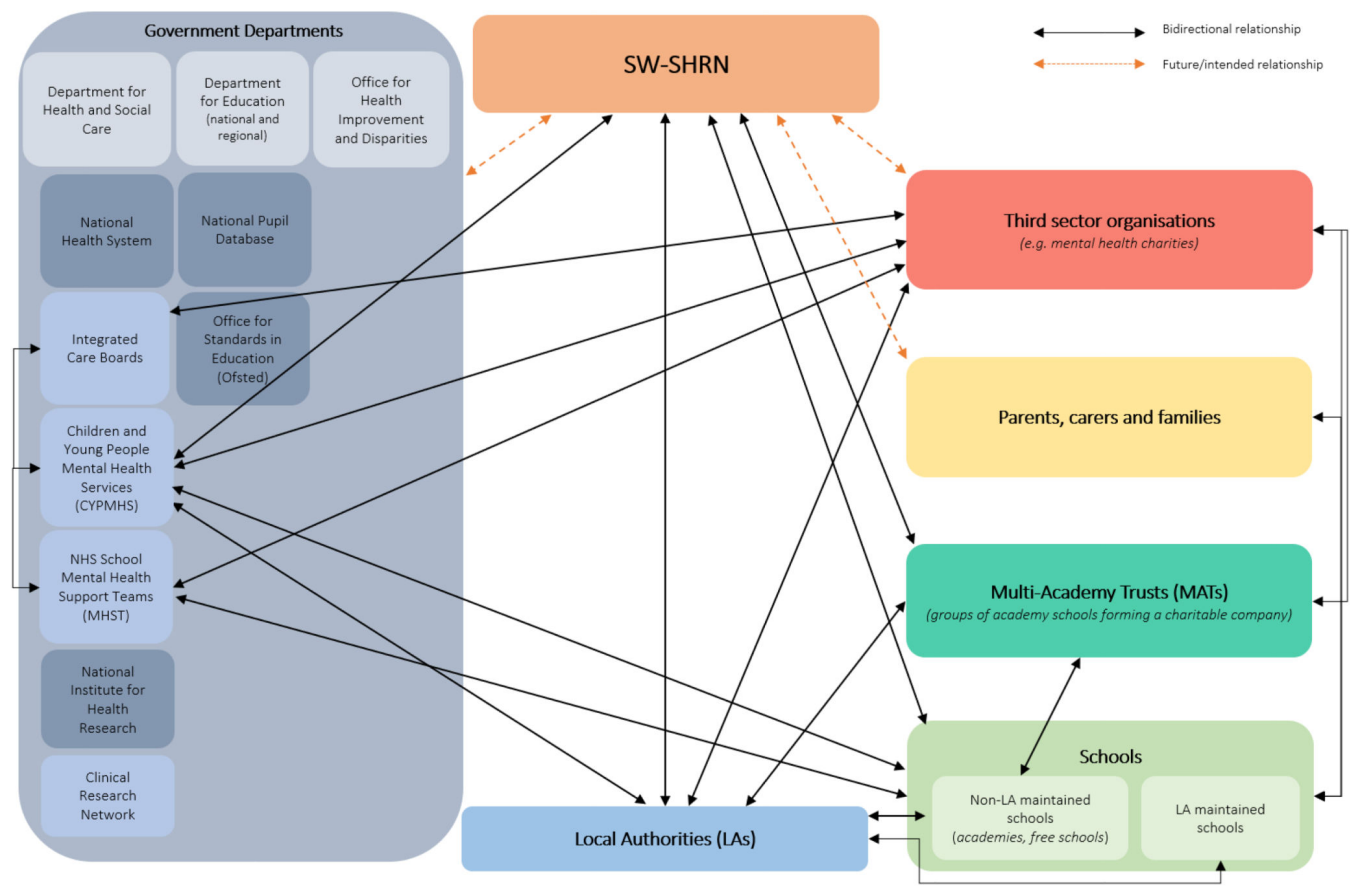
A working school systems map for England Figure 1 shows our working systems map, which depicts how the SW-SHRN operates within the English education system. With the network as the central point of the map, we have bidirectional relationships (depicted by the black arrows) with schools, Multi-Academy Trusts (MATs), Local Authorities (LAs), and various government departments. In future, the network hopes to establish bidirectional relationships (shown with orange, dotted arrows) with third sector organisations (such as mental health charities), families and carers of the school students, and other governmental departments, for instance the Office for Health Improvement and Disparities (OHID) or the National Pupil Database. This figure also shows the bidirectional relationships between the various actors in this map, for example, LAs with Schools, MATs, and third sector organisations.

This paper aims to use a systems perspective to evaluate the South West - School Health Research Network (SW-SHRN) (Sharp et al., 2022) using National Institute of Health Research, School for Public Health Research (NIHR SPHR) Guidance on systems approaches (Egan et al., 2019). In line with the Medical Research Council’s 2021 framework for developing and evaluating complex interventions (Skivington et al., 2021), this paper aims to:

1. Better understand the wider system that school health research networks operate in (educational, health and political)
  1.1. Further inform and refine our working school systems map for England (Figure 1)
2. Understand how school health research networks could contribute to system change
3. Understand how evidence generated from school health research networks can be used to support decision making and policy planning.

## Method

### Study design and participants

This process evaluation forms part of a larger pilot study of the SW-SHRN in which secondary school students aged 12-15 (n=5,211) participated in a health and well-being survey (Sharp et al., 2022). The survey topics include mental health and well-being, physical activity and eating behaviour, sexual health, risky behaviours including smoking and alcohol use, body image, sleep, peer support, cyberbullying, social media use and the school connectedness (Sharp et al., 2022). Participating schools (n=18) and local authorities (n=7) received tailored feedback reports on the student survey data and researchers worked closely with schools to identify key areas of focus for health improvement and to facilitate sharing of best practice between schools across the South West of England.

The process evaluation of this pilot study was divided into three components: 1) advice and recommendations for working with schools to conduct health research; 2) barriers and facilitators to implementing the SW-SHRN; and 3) using systems thinking to evaluate SW-SHRN. This paper focuses on using systems thinking to understand how the SW-SHRN operates within wider educational and health systems. Our write-up follows the Consolidated criteria for reporting qualitative research (COREQ) reporting guidelines (Tong et al., 2007).

Interview participants comprised school staff (n=11), local authority members (n=5), and wider key stakeholders (n=10), including policy makers, governors, academics, charity leaders and school-based National Health Service (NHS) staff.

### Data collection

Interviews took place between September 2021 and July 2022 within the context of the Covid-19 pandemic, however there were no pandemic-related restrictions in place at the time of the interviews. A question regarding the network and its potential impact to help schools respond to the pandemic was included in the interview topic guide and the research team anticipated that schools may have a heightened interest in student health and well-being as a result of the pandemic.

All participating schools had a lead contact who were all invited to be interviewed and participating and non-participating local authorities were invited to interview. Key stakeholders were identified by the research team at the study outset, all key stakeholders worked within children and young people’s health and well-being. 38 potential participants were contacted via email by a member of the study team, of who 26 participated in an interview. Of the 12 participants who declined, 9 did not respond to the initial email, 1 declined due to lack of capacity, 1 declined due to no longer working in young people’s health and 1 declined due a concern that by agreeing to interview their organisation might be seen as endorsing the network.

Semi-structured interviews were conducted by EW, a female public health researcher with experience in qualitative and mental health research in schools. Interviews ranged from 22 minutes to 67 minutes in length and took place remotely either via telephone or online platform (e.g. Microsoft Teams) with just the researcher and the interviewee present. Prior to the interview, all participants received a full information sheet and consent form. The information sheet contained an overview of the aims of SW-SHRN, a description of the qualitative work and why they had been invited to be interviewed and information on withdrawal, data confidentiality and what will happen to the results of the study. If written consent was not received before the interview took place, verbal consent was taken (and recorded) before the interview began. EW had no prior relationship with any interviewees ahead of study commencement. School and local authority staff knew the interviewer through their participation in SW-SHRN, stakeholders without any existing knowledge of the study were sent a description of SW-SHRN and EW introduced herself and the purposes of the study at the beginning of each interview.

Interviews followed two topic guides (see supplementary materials), the local authority/stakeholder topic guide included questions on stakeholder views on the network, their perceived barriers and facilitators, what outputs they would like to see from the network and how to make the network sustainable and scalable. The school staff topic guide included questions on school recruitment methods, experiences of participation, feedback on administering the student survey, views on tailored school reports, how they would use the data provided with their school and what would encourage them to continue being part of the network. The topic guides were circulated among academic and public health colleagues for feedback prior to use.

Ethical approval for the study was granted by University of Bristol’s Faculty of Health Sciences Research Ethics Committee (Ref. 110922).

### Data analysis

A framework approach was used to analyse the interview data given its ability to incorporate deductive analysis as well as allowing for inductive analysis (Gale et al., 2013). Codes were both deductive (generated from the topic guide) and inductive (generated from interview data). The following seven steps were observed:

1) **Transcription:** Audio recordings were transcribed verbatim, reviewed, and checked for accuracy by EW prior to analysis. All transcripts were initially read by EW to gain familiarity with the data.
2) **Familiarisation:** To continue familiarisation with the data, two researchers (EW and LH) independently read six transcripts; two school contact interviews, two local authority member interviews and two wider key stakeholder interviews.
3) **Coding:** EW and LH then independently annotated these 6 transcripts to generate an initial list of codes and draft framework.
4) **Developing a working analytical framework:** EW and LH then met to discuss and compare these initial codes and agree on a final set of codes to apply to all subsequent transcripts in order to create the analytical framework.
5) **Applying the analytic framework:** Although there were some distinct differences between school contact interviews compared to wider stakeholders, there was sufficient overlap to allow all transcripts to be coded using the same analytical framework. Subsequent transcripts were single coded by either EW or LH, applying the agreed analytical framework, with further regular discussions to clarify or expand the framework as needed.
6) **Charting data into the framework matrix:** EW and LH then charted the data into the framework matrix by creating summaries and identifying key quotes for each category (grouped codes).
7) **Interpreting the data:** EW and LH then met regularly to interpret the data, mapping connections between categories, identifying central characteristics and comparing data categories between and within cases to generate a set of themes and subthemes. Themes and subthemes were then discussed, revised and agreed by all co-authors.

NVivo qualitative data analysis software package (QSR International, version 12) was used to aid data management.

## Results

Table 1 provides a summary of interview participants by organisation type, role type and gender.

### Theme 1: Fitting in with the wider landscape

A central theme throughout the interviews was the importance of SW-SHRN not being a siloed system and the need for the network to become meaningfully embedded within existing work on young people’s health and well-being across England. Stakeholders acknowledged that there was a lot of activity around children and young people’s mental health in schools and highlighted the importance of addressing gaps and aligning with ongoing practice and avoiding reinventing the wheel. This theme was broken down into four subthemes.

#### 1.1 Complexity of England’s education system: a key challenge for systems-based approaches

The first subtheme acknowledged the complexity of the school system in England. This was primarily discussed in relation to recruiting schools to the network and recognising the need to form close relationships with local authorities, multi-academy trusts but also individual schools. Stakeholders discussed how local authorities used to act as a central point of contact for schools, but that there was now substantial variation between schools regarding their relationship with local authorities and no sub-national strategic approach to health promotion in schools. For example some academy schools have little to no contact with local authorities and have their own independent health promotion strategies.

“I think, getting into schools has become a lot more challenging just because of how they’re set up and the academy structure. It used to be that you could go through the local authority and have a quite straightforward way of getting into schools because they were quite linked up with what they were doing. I think now that...has broken down and… it’s very much up to the individual schools, or certainly the academies, whether they want to engage or not.” (KS 6)

“There’s variation across the region, in the strength of the relationship the local authorities have with their local schools and academies. Previously, local authorities have been the main stakeholder and gatekeeper for health promotion and a strategic approach...to mental health and wellbeing. With that change towards a more autonomous system for some of the schools, it’s not so easy.” (KS 4)

“Local authorities are really valuable, but there are areas where 90%+ schools are academized. So if you’re just working with the local authority, it’s a tiny amount of information that you’re getting. They do have some contact obviously with Multi-Academy trusts, but the trusts are very independent and they can take or leave advice. They are very much independent bodies and they’re all very different.” (KS 10)

Given the variation of health promotion efforts across schools in England, local authorities discussed the benefits of a network offering a coordinated approach to reduce duplication of similar work as well as the need for focussed efforts on school health improvement due to reduction in resources within local authorities.

“We could save time and energy having a coordinated approach. So, then, yes, I think networks of schools coming together to share good practice is useful. We’re all essentially doing the same work, and local authorities have been quite depleted in terms of staff, particularly school improvement advisors. So, we no longer have the resources to provide all the school improvement activities that we once did. So, there’s certainly room in the marketplace for many providers, and I think it would allow local authorities to perhaps focus more on different things that aren’t provided elsewhere.” (LA 5)

#### 1.2 Addressing changing needs and political interest

Key stakeholders, particularly those working in Government departments, emphasised the importance of school health research networks offering some targeted work to address unmet need with a particular focus on addressing health inequalities. Politics and changing political interests relating to health were also discussed with reference to the importance of demonstrating meaningful change and the network being capable of demonstrating meaningful action. One stakeholder highlighted the need for change to move beyond individual behaviour change to address wider societal impacts on health.

“Currently, I’d say… the levelling up agenda [focus on improving lives in areas of the UK which have historically received lower funds] … addressing inequalities… you target your resources where it’s most needed whilst also having one eye on what the general population needs. But you must have an explicit way to demonstrate that this closes the gap… to address inequalities. You need to be able to demonstrate explicit links to outcomes that are deemed to be of interest, politically… that changes over time…so having, a logic model behind it that demonstrates those pathways.” (KS 3)

“It’s politics, you know? With health improvement it’s long term…we’ve got these widening health inequalities, what do we know that will fix that, and what are we doing based on that? How do we address those entrenched problems and challenges to our [young people’s] health, like obesity, smoking problems, physical activity levels and readiness to learn, those kinds of things? That won’t be effectively addressed by just focusing on individual behaviours and telling children and young people what they should be doing.” (KS 4)

#### 1.3 Linking with existing networks, services and intervention providers

Stakeholders highlighted the importance of the network not being a siloed system. They indicated a needs for the SW-SHRN to be embedded within the wider national picture of school-based health and well-being and to take account of the existing work in this area if it was it to be meaningful and valued over time. This included similar networks as well as NHS services (Children and Adolescent Mental Health Service) and other local and national approaches.

“It is thinking, “How do we make sure that any network in the South West is also linked into other networks and is doing things that are complementary with other networks?” You know, we are not the only people having this network.” (KS 7)

“How will they [schools] then link [network findings] in with the system, with CAMHS (Children and Adolescent Mental Health Service) or whatever the local system is or the national system? How will they then take that next step? So, they’ve been told that a large number of people in Year 10 have got some kind of issue with anxiety, then what are they going to do next, that links into the wider system? Because I guess what you’re looking for is system change.” (KS 1)

One stakeholder also discussed which areas are in need of support given the context of existing priorities. For example, there is currently a large focus on areas of high deprivation, but adolescents attending schools in more affluent areas are also reporting increased mental health difficulties and this may be less of a focus, suggesting a potential change in which areas could most benefit from a new network. This idea again reflected on SW-SHRN operating within a wider landscape and not repeating existing efforts.

“It’s finding something that’s not already 1) that they’ve already bought in to, or 2) being offered...those schools that work with mental health support teams, part of the mental health support teams’ role is to do assemblies, staff training sessions, all of those kind of things, with a focus on mental health, but also more broadly health and wellbeing and behaviour, because that all feeds in. So there are quite a lot of schools with access to that kind of thing, but it’s inconsistent. And I think a new offer would need to be targeted to particular areas or need…also at the moment…it’s being prioritised in areas of high deprivation, or it’s all being done on need indices. So there are big groups of schools that are in more affluent areas where mental health and wellbeing and health is still a really important problem, but they are the areas that are not getting so much support at the moment.” (KS 10)

#### 1.4 Regional vs. national network

Stakeholders discussed the strengths and weaknesses of a regional versus national networks. Strengths of a regional model included tailored provision, a richer local picture, and shared practice on a regional level, which was highlighted by one stakeholder as a current gap.

“I mean, at the moment, in terms of children’s health and wellbeing, clearly, we’ve got the health behaviour in school aged children survey that runs every four years. But that only generates data at a national level and not at a local or regional level. And so the opportunity to act on that in, sort of, meaningful ways that connect with local communities and schools is limited.” (KS 3)

However, many stakeholders also work on a national level, particularly with regards to national policy and national children and young people databases. Stakeholders noted that, in order to drive national policy, the network would need to evidence benefits beyond the South West. Although, some stakeholders stated that we have existing networks generating national data, and they can make it difficult to act locally and in a more targeted way.

“I think there is a question in there as well about how you grow the data to a national data set.

People seem to come with this idea of schools measuring wellbeing for two goals. And one is school improvement...but the other side is we need better data on children’s health and wellbeing. And if we measure through schools, you can get both of those things...if they’re all measuring consistently, you can flow up into a huge national data set, as they have in Wales now. I think, at national level, there is a potential for just richer data on health and wellbeing.” (KS 2)

“So for me, the question is how does what’s happening in the Southwest region around this agenda offer anything that has wider benefits beyond just the Southwest?” (KS 3)

### Theme 2: Partnership working

A key message from stakeholders was the importance of connecting individuals and organisations involved in young people’s health and well-being. Our existing stakeholders (government departments, charities, NHS initiatives, local authorities and academy governors) provided a number of suggestions for partnership working between the network and other individuals or organisations.

#### 2.1 Representation in school

A number of suggested key partnerships for SW-SHRN involved working closely with ‘on the ground’ health staff in schools, for example SW-SHRN data could be shared and discussed with school nurses to create and implement resources in the identified areas of need.

“One of my hobbyhorses [issues] when people are working in schools is to remember school nurses. I know they are in some ways quite a scarce resource but they’re always there in the background. One of our desires…is to make the public health role of school nurses much more evident than maybe it has been and the desire if you look at the commissioning guidance around school nursing. So, my advice would be to try and ensure that you link…with what the local school nurse provider is doing…because sometimes they’re closer to the school structure and what’s happening than the local authority is just because they’re in the schools.” (KS 6)

Teaming up with existing initiatives was also suggested, for example linking in with the ongoing work of NHS school Mental Health Support Teams (MHSTs) and sharing the data to inform more targeted support.

“Our model has an identified mental health lead in the school we work with, our go-to person, if they’ve got access to [the networks] benchmarking data and they can see their year sevens have got a really high number of children who are showing high GAD7 [anxiety], then we think, “Right, we need to put something into the year seven group.” There’s so much scope for working with [mental health support teams] because you know this is a whole school approach.” (KS 9)

Stakeholders touched on the value of having a permanent/named person who understands the local context and whose role is to respond to needs that are identified from the SW-SHRN survey data.

“You need a constant learning team, to be reacting, to support our more statutory, established education. So, a team of people who can create resources...that can implement resources locally that are reactive to the local need, or what is happening at that moment in time. They need to be permanently in post”. (LA 1)

#### 2.2 Ofsted: opportunities and challenges

Some stakeholders suggested a potential future partnership with Ofsted (Office for Standards in Education, Children’s Services and Skills) could be beneficial with Ofsted making use of the network data to inform local area inspections. Ofsted is a government organisation in England that carries out inspections to grade schools on their quality and performance. Schools are placed into one of four categories; Grade one (outstanding), grade two (good), grade three (requires improvement) and grade four (inadequate). After inspections, Ofsted publish their findings so they can be used to improve standards in education.

“I think Ofsted should be a stakeholder...it’s just finding and navigating to the people who manage the data, who use the data to inform area level inspections and why they would be interested. They have been interested in the past, in our national fingertips profiles to read and inform what they inspect at an area level, local area inspections. So, I think making contact with and nurturing a relationship and inviting them to contribute to be informed about this network, would be really important.” (KS 3)

However, this idea was somewhat at variance with the perceived benefit of having a university leading the network and the trustworthiness and impartiality which that implied.

“Having an external organisation makes it more credible for the students, and the schools, who might, I think, wrongly suspect that the council, I don’t know, producing-holding data and might be using it in ways that they might not agree with, or fiddling the data, to some extent.” (LA 5)

Ofsted also detailed that they would be unable to publicly endorse the network or any particular model and typically only report on what is working well in certain areas. This is to avoid schools thinking they must take part in certain initiatives, which could take additional time, money, or may be a burden on schools. Additionally, there was added concern with government departments holding data due to power imbalances and potential misuse of data. Therefore an Ofsted partnership would need careful consideration.

“The big one is the accountability risk. If government generates this school level-identifiable data about every school...there is a risk that the data gets published and people make league tables out of it, even if that’s not what it’s intended for. So, there is that kind of risk, I think, of government having that data. Government departments have a reputation and a power and there is a power imbalance there, that feels like it wouldn’t be a good partnership with schools, collecting and creating the data.” (KS 2)

#### 2.3 Sharing data and practice across systems

Stakeholders offered advice on how to work effectively with key partners and how ‘share data, good practice and key learning from the network across the system. They stressed the importance of placing the findings within the wider evidence base and using SW-SHRN as a platform to share existing policy and practice as well as sharing network-specific findings.

“Just sharing what policies and things they have in place already. So, what is working in different areas, so they could actually utilise [policies] in their own schools or an area that they’re not doing at the moment that might be helpful to them. So, it’s just...sharing good practice, isn’t it. I think that’s really important, and particularly if schools...would like to do more of that themselves, like peer support. So, if someone’s got a particular issue in their school, they could look in the network and see, “Well, actually, this area of health is really terrible in this school. What can we do about it?” and they could ask other people in the network.” (KS 5)

“If it is a living network, that it is really working collaboratively and sharing any learning successfully across other systems.” (KS 1)

Stakeholders also discussed the usefulness of having shareable datasets to allow direct access to anonymised data, particularly for analyses to inform policy.

“If there are big data sets that are produced, access to them. Not necessarily identifiable data, you know, like in a usual data-sharing way, for our own research and analysis. And the evidence that others could produce by analysing those, so there is that evidence for national policy, I think would be the big thing. Ultimately, I would like shareable data sets, obviously in a sensibly secure sharing environment. So, research data sets.” (KS 2)

Schools learning from one another and learning what works in particular health areas was seen as key, particularly if schools could see a worked example or case study of the process of using SW-SHRN data to make a change which then led to improved health outcomes.

“I think schools can always learn from other schools. I think when we know, in one particular school... their students are a lot more mentally healthy, we need to look at those schools, and see what they’re doing, and see what we’re not doing. I think that does help...knowing that you’re not getting

it, like, “The students aren’t 100% in this area, but in another school they are, so what are they doing, and how can we compare? How can we use each other to support our students?” I think is really important.” (SC 15)

“Obviously, every school is different, but if they can see it’s been beneficial in how a school has grown or shifted their culture or something like that. So it doesn’t feel like, “Oh, here are just some more people wanting to audit us and tell us what to do.” But actually, “We went through this process and this is how things have changed and there’s been an improvement,” is a really valuable thing to hear.” (KS 10)

### Theme 3: Informing policy and allocation of resources

#### 3.1 Informing policy and guidance

Key stakeholders noted the importance of large datasets for informing local and national policy and guidance, but particularly noted the strength of local/regional data to inform local priorities and to update both local authority guidance and individual school policies.

“I think the risk from people in a strategic role, we’re kind of midstream. We’re more upstream than at a local priority level. We’re working closer to the department and to ministers, and it [network data] will provide us with a bit more of a solid picture to present, about how policy and interventions are likely to land in local schools. Sat behind a desk in Whitehall, or wherever, churning out policy documents or strategy is one thing, but it needs to be informed by who is going to receive it, and who is going to work with it.” (KS 4)

“If we can see that there are issues that have caught up in the questionnaire, then that will obviously galvanise us into thinking about how we can then influence policies. We have certain documents anyway, which are guidance documents for schools, and it may well be that they do get revised in the light of the findings…So I think, actually, that there is quite a lot of potential for just changing the kind of guidance, sample policies and things we give to schools, definitely.” (LA 2)

#### 3.2 Distribution of funding and resources

Stakeholders, particularly local authorities, detailed the usefulness of SW-SHRN data in terms of better distribution of funding and allocating resources in known areas of need, which links with an earlier point around targeting network activity based on existing practice and underserved communities.

“If we knew there were particular concerns in the South West around eating disorders, then we could focus quite a lot of our work on that and we could seek funding in that area. So, it is really enormously helpful to have that information, because then it can influence what we are going to do and the funding that we seek and what we put our priorities on as well. And that would be the same for anyone, for CCGs, for all areas.” (KS 1)

**Figure 3:**
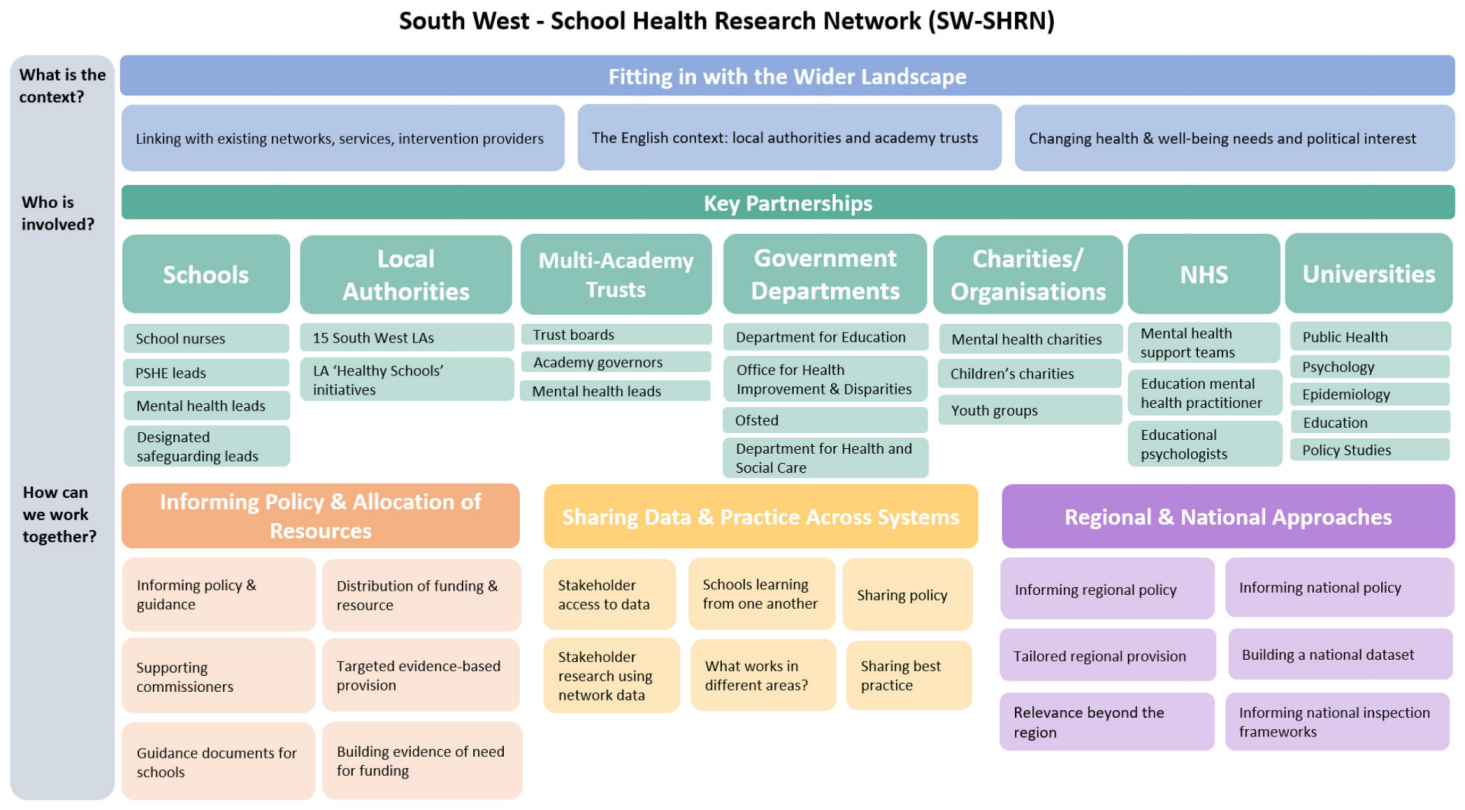
A working model of SW-SHRN based on the main themes and subthemes

To address the aims of this process evaluation, Figure 3 maps out the themes and subthemes onto a working model of how SW-SHRN operates which illustrates a number of ways school health research networks can contribute to system change and be used to support decision making and policy planning. We are also hopeful that in the future we will be able to conduct some further systems mapping work in light of these findings to refine and develop our working systems map (Figure 1).

## Discussion

The interviewees in this study highlighted the importance of the SW-SHRN linking to other elements of the educational infrastructure to make sure that it did not become a siloed system. Stakeholders consistently underscored the importance of ensuring that school health research networks are meaningfully embedded within existing health and well-being policy and practice in England. Stakeholders also stressed the need to maintain and strengthen multi-sectoral partnership working as this is needed to facilitate continuous knowledge sharing between key partners, and drive national policy and planning.

Key themes identified in this study are consistent with evidence from other system-based health evaluations finding that strong relationships between stakeholders, allowing time to build relationships, trust and local/community capacity, embedding initiatives in a broader policy context and ensuring the approach is robust and sustainable are all key to building a successful whole systems approach (Garside et al., 2010, Bagnall et al., 2019).

The value of sharing ‘what works’ among schools was another important finding, with recommendations to share success stories, present health area-specific case studies and highlight best practice to participating schools within the network. As well as sharing what works among schools, school staff, local authorities and wider stakeholders all requested that the networks findings were embedded into existing policy and evidence to make them more meaningful for both policy and intervention planning. Rather than giving schools a static report of findings, researchers could play a role in interpreting the findings in line with existing policy and evidence to provide policy briefings and evidence summaries in lay terms for schools and local authorities. This illustrates the multiple functions of SW-SHRN, working at different levels across the system, e.g. highlighting areas of focus for individual schools, indicating areas to target provision for local authorities and providing up to date evidence of adolescent mental health for wider policy planning. Providing up to date data as it becomes available in the form of ‘feedback loops’ has been shown to be effective in previous whole school approaches allowing data to continuously inform activity and allow for responsive and continuous improvement (Kearney et al., 2016).

An important finding in relation to future school inspection frameworks and an increasing focus on health and well-being data within schools, was schools’ concerns around being judged or ranked on their health data and the existing power imbalances between government bodies and schools. Many stakeholders believed it would be important to assess schools on their engagement in promoting health and well-being in schools, rather than any comparison between health and well-being outcomes. Ofsted is likely to play a key role in health and well-being promotion in schools in the future and could be an important area of future growth of the network, however this relationship needs to be more closely evaluated. Although the Welsh SHRN model work closely with Ofsted (Estyn in Wales) we are yet to see evidence to support the pros and cons of this partnership.

Understanding different stakeholder perspectives regarding the delivery of SW-SHRN and the use of SW-SHRN data, enabled us to identify strategies for intervention sustainability and scalability. Strategies for success centred around partnership working at multiple levels and embedding network activity within national and local policy. Stakeholders noted the strengths of offering both tailored local resource targeting whilst providing data to inform national policy and planning. Network findings allow data-informed planning for schools, local authorities and policy makers.

Future research should aim to gather deeper insights into more components of the health and education system in England to more formally map out the system in which SW-SHRN sits and examine where and how school health research networks can fit into this system.

### Strengths and limitations

This study provides unique insight into applying systems thinking to evaluating school health research networks, a novel evaluation that is yet to be carried out on other existing networks. This study also benefits from seeking perspectives from a wide range of stakeholders, multiple local authority areas and a variety of school staff from eleven individual schools. However, the following limitations must also be acknowledged. There are several types of school that did not participate in the SW-SHRN pilot including free schools, faith schools and independent schools, therefore school staff perspectives from these different school types may differ to our reported findings. Equally, members from only 5 of a total of 15 local authorities in South West England were interviewed as part of this process evaluation and therefore local authority viewpoints are not necessarily representative of the whole of the South West region. It will be important to run further qualitative work if additional school types and local authorities are recruited to the network in future. Further recruitment and expansion of SW-SHRN is therefore required to gain a more in-depth understanding of the regional system and gain perspectives of the other local authorities in terms of how their structures operate. Additionally, there is potential for such networks to improve the health and well-being of staff as well as students, however the SW-SHRN pilot study did not collect staff health and well-being data, which could be another important area for future expansion.

## Conclusion

This qualitative process evaluation details the barriers and facilitators to implementing a regional school health research network to improve adolescent health and well-being. The findings highlight the vital nature of multi-sector partnership working and intervening and monitoring at multiple levels.. The findings also draw attention to the complexity of the current landscape of school health promotion in England and the need to take a systems approach. School health research networks can play a key role in facilitating the needs within the system. These include collaboration, identifying local need, better targeting of resources, sharing data and best practice across different parts of the system and informing regional and national policy.

## Supplementary Material

Supplementary Material

## Figures and Tables

**Figure 2:**
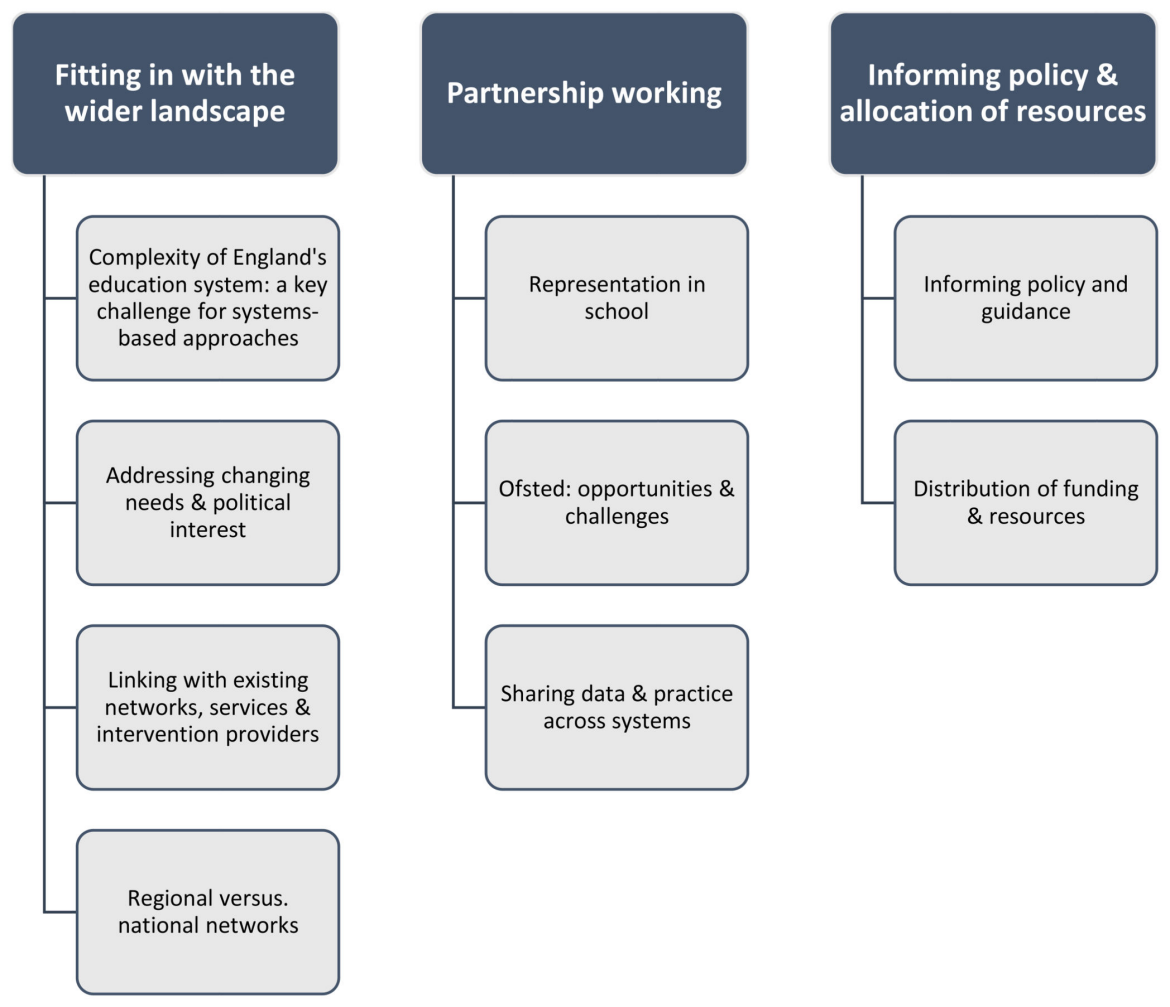
Diagram of Qualitative Themes and Subthemes. Three main themes were developed from the framework analysis; 1) Fitting in with the wider landscape; 2) Partnership working; 3) Informing policy and allocation of resources. The key themes and subthemes are summarised in Figure 2.

**Table 1:** Summary of Key Stakeholder Interviews by Organisation, Role Type, and Gender

Interview	Organisation Type	Role Type	Gender
KS1	Charity	Mental health lead	Female
KS2	Government department	Mental health, national	Female
KS3	Government department	Public health, national	Female
KS4	Government department	Public health, regional	Male
KS5	Government department	Research lead, national	Female
KS6	Government department	Public health, national	Female
KS7	University	Clinical Psychologist/Academic	Female
KS8	Academy Trust	Governor	Male
KS9	NHS	Mental Health Support Team	Female
KS10	Government department	Mental health, regional	Female
LA1	Local authority	Advanced Public Health Practitioner, Health & Well-being	Female
LA2	Local authority	Health Improvement Specialist: Children & Young People	Male
LA3	Local authority	Children & Families Commissioning	Male
LA4	Local authority	Lead for Health and Well-being	Female
LA5	Local authority	Children and Families	Male
SC1	Participating school	Deputy Head Teacher	Female
SC2	Participating school	Pastoral Support Worker	Female
SC3	Participating school	Deputy Head Teacher	Female
SC4	Participating school	Head of Personal Development Curriculum	Female
SC5	Participating school	Deputy Head Teacher, Student Welfare & Behaviour	Female
SC6	Participating school	Music Teacher, Lead for Looked After Children	Male
SC7	Participating school	Mental Health & Well-being Coordinator	Male
SC8	Participating school	PSHE Lead	Female
SC9	Participating school	Assistant Headteacher	Female
SC10	Participating school	Deputy of Physical Education and Health, Personal, Social Health & Economic education Lead	Female
SC11	Participating school	Deputy Head Teacher	Female

‘KS’ = key stakeholder, ‘LA’ = Local Authority ‘SC’ = School Contact

## Data Availability

The datasets used and analysed during the current study are available from the University of Bristol data archive, https://data.bris.ac.uk/data/.
